# Mutation of the Xylanase regulator 1 causes a glucose blind hydrolase expressing phenotype in industrially used *Trichoderma* strains

**DOI:** 10.1186/1754-6834-6-62

**Published:** 2013-05-02

**Authors:** Christian Derntl, Loreta Gudynaite-Savitch, Sophie Calixte, Theresa White, Robert L Mach, Astrid R Mach-Aigner

**Affiliations:** 1 Department for Biotechnology and Microbiology, Institute of Chemical Engineering, Vienna University of Technology, Gumpendorfer Str. 1a, Wien, A-1060, Austria; 2 Iogen Corp., 310 Hunt Club Rd., Ottawa, ON, K1V 1C1, Canada

**Keywords:** *Trichoderma reesei*, *Hypocrea jecorina*, Cellulases and hemicellulases, Inducer-independent enzyme production, Biofuel, Xylanase regulator 1 (Xyr1), Glucose response domain

## Abstract

**Background:**

*Trichoderma reesei* is an organism involved in degradation of (hemi)cellulosic biomass. Consequently, the corresponding enzymes are commonly used in different types of industries, and recently gained considerable importance for production of second-generation biofuel. Many industrial *T. reesei* strains currently in use are derived from strain Rut-C30, in which cellulase and hemicellulase expression is released from carbon catabolite repression. Nevertheless, inducing substances are still necessary for a satisfactory amount of protein formation.

**Results:**

Here, we report on a *T. reesei* strain, which exhibits a very high level of xylanase expression regardless if inducing substances (e.g. D-xylose, xylobiose) are used. We found that a single point mutation in the gene encoding the Xylanase regulator 1 (Xyr1) is responsible for this strong deregulation of endo-xylanase expression and, moreover, a highly elevated basal level of cellulase expression. This point mutation is localized in a domain that is common in binuclear zinc cluster transcription factors. Only the use of sophorose as inducer still leads to a slight induction of cellulase expression. Under all tested conditions, the formation of *cbh1* and *cbh2* transcript level strictly follows the transcript levels of *xyr1*. The correlation of *xyr1* transcript levels and *cbh1*/*cbh2* transcript levels and also their inducibility via sophorose is not restricted to this strain, but occurs in all ancestor strains up to the wild-type QM6a.

**Conclusions:**

Engineering a key transcription factor of a target regulon seems to be a promising strategy in order to increase enzymes yields independent of the used substrate or inducer. The regulatory domain where the described mutation is located is certainly an interesting research target for all organisms that also depend so far on certain inducing conditions.

## Background

*Trichoderma reesei* (telomorph, *Hyprocrea jecorina*) [1] is a filamentous ascomycete thriving as a saprophyte on dead plant material. It degrades cellulose and hemicelluloses by secreting a wide array of cellulases and hemicellulases. A genome-wide analysis revealed 10 celluloytic and 16 xylanolytic enzyme-encoding genes in *T. reesei*[2]. The most abundantly secreted and industrially interesting enzymes are the two main cellobiohydrolases, CBHI and CBHII (EC 3.2.1.91) [3], and two major specific endo-ß-1,4-xylanases, XYNI and XYNII (EC.3.2.1.8) [4]. We will use the term major, industrially relevant hemicellulases and cellulases (MIHCs) throughout this publication for these two cellulases and two hemicellulases.

The MIHCs work together with further enzymes to degrade cellulose and xylan. This results in the formation of soluble oligo- and monosaccharides, such as cellobiose, D-glucose, xylobiose, and D-xylose. In addition, sophorose is a product of the transglycosylation activity of some of these enzymes [5]. All of these molecules were reported to have influence on the expression of MIHCs in *T. reesei*. The presence of D-glucose causes carbon catabolite repression (CCR), which results in the secretion of low quantities of MIHCs; expression of XYNI is even completely shut off. Sophorose is the most potent inducer for the expression of CBHI and CBHII. It also triggers the expression of XYNII (reviewed by [6]). D-xylose modulates XYNI and XYNII expression in a concentration-dependent manner. Strongest induction occurs by using low concentrations (0.5 mM), whereas high concentrations lead to a repressing effect on xylanase expression [7].

Despite the different patter of inducibility of the expression of MIHCs, it generally depends on the presence of the main transactivator of hydrolases Xyr1 (Xylanase regulator 1). Xyr1 has a Gal4-like Zn_2_Cys_6_ binuclear cluster domain, which is involved in DNA-binding. A *xyr1* deletion mutant does not produce any MIHCs at the level of either transcription or of protein formation [8,9]. It has been reported that using D-xylose or xylobiose could not induce the expression of *xyr1* itself, even if these saccharides are potent inducers of XYNI and XYNII expression mediated via Xyr1. However, analogous to its target genes, Xyr1 expression is regulated by CCR mediated by Cre1 [10]. Cre1 is a wide-domain regulator that binds under repressing conductions (high concentrations of easily utilisable monosaccharides such as D-glucose or D-xylose) to its binding site in the promoter of e.g. *xyr1* or *xyn1* resulting in a down-regulation or a complete shut-off of transcription, respectively [11-13].

It is evident that a release from CCR is a useful prerequisite for industrial exploitation of *T. reesei* for the production of MIHCs. Therefore, a prominent Cre1-deficient mutant strain of *T. reesei*, Rut-C30, which was described as a high yielding cellulase mutant [14], has been used as the progenitor strain for many recent industrial strains. However, industrially satisfying production of MIHCs by Rut-C30 and its industrially used offspring still depends on potent induction. Using certain inducing compounds or a particular media composition is the common way to achieve this induction, but both add cost which may lead to a higher price for the resulting enzyme products. In particular, the economical, ecological and socio-economical success of products such as second-generation biofuel strongly depend on their cheap availability of MIHCs as well as a production process using non-food biomass as starting material.

In this study we report on an industrially used *T. reesei* strain that produces high amounts of MIHCs independent of the presence of a certain inducer. Moreover, this strain shows a glucose-blind phenotype when it comes to expression of MIHCs. Consequently, we analysed this strain at the transcriptional level in order to identify the molecular mechanisms behind this phenotype. Interestingly, we found aside to other mutations a single point mutation in Xyr1 and investigated to which extent this is responsible for the outstanding phenotype of that strain.

## Results

### A two-step mutant derived from *T. reesei* Rut-C30 yields elevated and aberrant xylanase expression due to a single point mutation in *xyr1*

**Figure 1:**
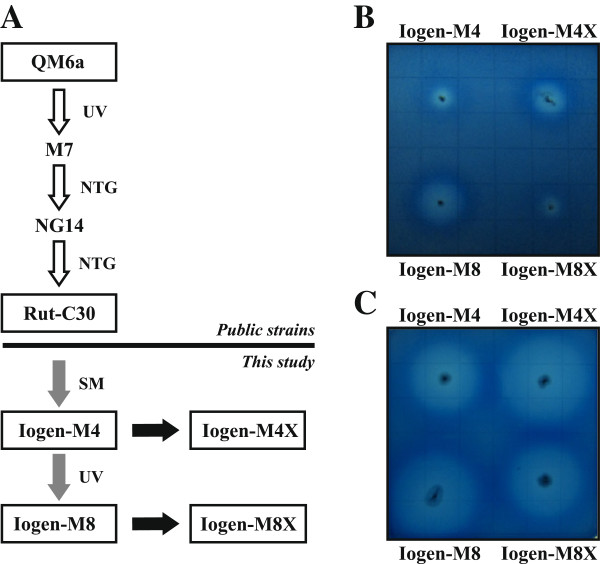
**Pedigree and xylanolytic properties of *T. reesei *strains used in this study.** (**A**) Schematic drawing of the pedigree of strains of which investigated ones are shown in boxes. Next to arrows the method of strain generation is indicated: UV-mutagenesis (UV), nitrosoguanidine (NTG), spontaneous mutation (SM). White arrows indicate strain generation before this study, grey arrows indicate strain generation during this study, black arrows indicate strains generated by targeted gene replacement during this study. Xylanolytic properties of strains generated during this study are represented by growth on plates containing 0.2% (w/v) azo-xylan as carbon source at 30°C for 24 h (**B**) and for 72 h followed by a 2 h incubation at 50°C (**C**).

The industrial strain Iogen-M4 (derived from Rut-C30 by spontaneous mutation) was used as parental strain for UV mutagenesis. During the subsequent screening process the mutant Iogen-M8 was selected due to its elevated xylanase activity. Cultivation in a bioreactor revealed a generally elevated protein secretion rate and confirmed an increase of xylanase expression compared to its parental strain. High levels of XYNI and XYNII could even be found in the supernatant when cellulase inducing conditions were applied. An additional figure presenting ELISA data shows this more in detail (see Additional file 1). In order to identify a possible genetic cause for this behaviour, the genomes of Iogen-M4 and Iogen-M8 were sequenced. The two strains differ in 17 point mutations within ORFs giving rise to mutations in known or predicted proteins. One of which is a point mutation within *xyr1* manifesting in the expression of an altered Xyr1 having alanine replaced by valine at position 824 (A824V). The complete sequences of the wild-type and mutated *xyr1* and Xyr1 is given in an additional file (see Additional file 2). This point mutation was introduced into Iogen-M4 resulting in the strain Iogen-M4X. This strain leads to an identical expression of cellulases and hemicellulases behaviour like Iogen-M8. *Vice versa*, re-establishing the wild-type *xyr1* in Iogen-M8 resulted in the strain Iogen-M8X exhibiting xylanase expression levels comparable to the parental strain Iogen-M4. Figure 1 gives an overview of these strains, their pedigree, and their xylanolytic properties when grown on xylan plates.

### Strong deregulation of expression of MIHCs can be observed in *T. reesei* Iogen-M8

The outstanding properties of Iogen-M8 with regard to xylanolytic activity were investigated on the level of transcription to identify changes in regulatory mechanisms of expression of MIHCs compared to Rut-C30, its first ancestor with a Cre1-negative background. Therefore, both strains were pre-grown in Mandels-Andreotti (MA) medium containing solely glycerol as carbon source. These mycelia were replaced into MA medium containing either 50 mM D-glucose, 0.5 mM D-xylose, 66 mM D-xylose, or 1.5 mM sophorose as carbon sources or inducers [7,15,16]. An additional culture was incubated in MA medium without carbon source as reference. Samples were taken directly from the pre-culture (before the replacement) and after three and six hours of incubation. RNA of these samples was extracted and used as template for RT-qPCR analysis.

**Figure 2:**
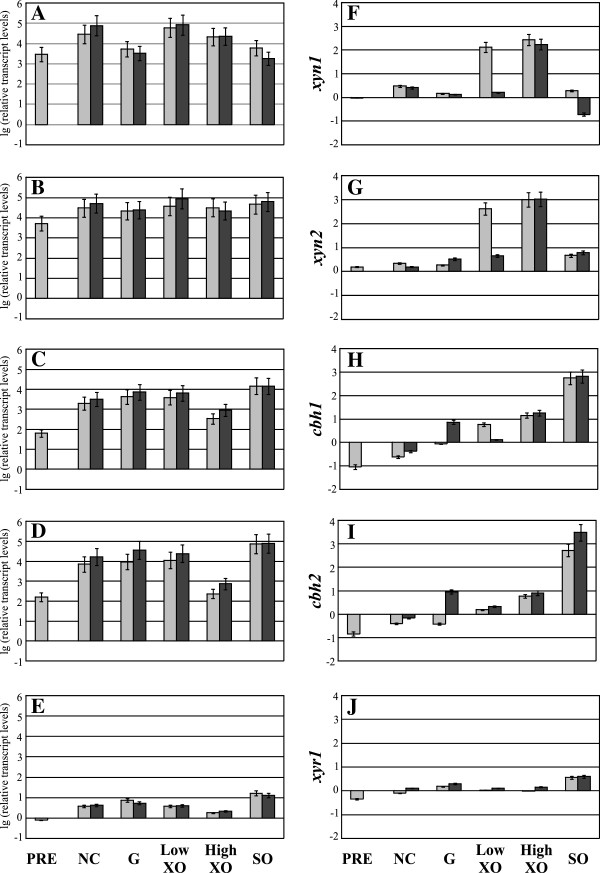
**Analysis of transcript levels of MIHCs-encoding genes and *xyr1 *in *T. reesei *RutC30 and Iogen-M8.** The xylanase overexpression strain Iogen-M8 (**A**-**E**) and its ancestor strain Rut-C30 (**F**-**J**) were precultured on glycerol and thereafter transferred to MA media without carbon source (NC) or containing 50 mM (w/v) glucose (**G**) or D-xylose (XO) at the indicated concentration, or 1.5 mM sophorose (SO) as an inducer. Samples were taken directly before transfer of mycelium (PRE), after 3 h of incubation (light grey bars), and after 6 h of incubation (dark grey bars). Transcription analyses of indicated genes from both strains were performed via qPCR using *sar1* and *act* transcript levels for normalization. Results are given as relative transcript ratios in logarithmic scale (lg). The values provided in the figures are means from three biological experiments. Error bars indicate standard deviations. Transcript levels always refer to the reference sample (wild-type QM6a, NC 3 h).

Transcript levels of *xyn1* and *xyn2* of Iogen-M8 could be detected at a constantly high level, regardless if any, or which, carbon source or inducer was used (Figure 2A, B). This finding correlates with the elevated and deregulated xylanolytic enzyme formation observed previously during the UV mutant screening procedure. Generally, Iogen-M8 shows considerably higher transcript levels of all tested genes under all conditions compared to Rut-C30 (compare Figure 2A-E to Figure 2F-J); e.g. transcript levels of *xyn1* and *xyn2* are 100- to 10,000-fold increased compared to those in Rut-C30 (compare Figure 2A to Figure 2F and Figure 2B to Figure 2G, respectively). In contrast, in Rut-C30, other than a general, slight deregulation, the genes *xyn1* and x*yn2* are both inducible by D-xylose (Figure 2F, G).

In addition, the transcript levels of the main cellobiohydrolase-encoding genes, *cbh1* and *cbh2,* showed a striking increase (up to 10,000-fold) in Iogen-M8 compared to Rut-C30 (compare Figure 2C, D to Figure 2H, I). These genes remain subject to induction in Iogen-M8 as a comparison of their transcript levels derived from incubation without carbon source to those with sophorose reveals (Figure 2C, D), even if the extent of inducibility is less pronounced than in Rut-C30 (Figure 2H, I). Notably, in both strains *xyr1* transcription is inducible by sophorose (Figure 2E, J), and the patterns of transcript levels of *cbh1* and *cbh2* reflect those of *xyr1* on all carbon sources or inducers tested (Figure 2C-E and Figure 2H-J).

In summary, Iogen-M8 has general highly elevated transcript levels of all genes under all investigated conditions compared to Rut-C30. Consequently, we conclude Iogen-M8 exhibits a strong deregulation in expression of MIHCs.

### Deregulation of MIHCs expression only partly occurs in the parental strain of Iogen-M8

**Figure 3:**
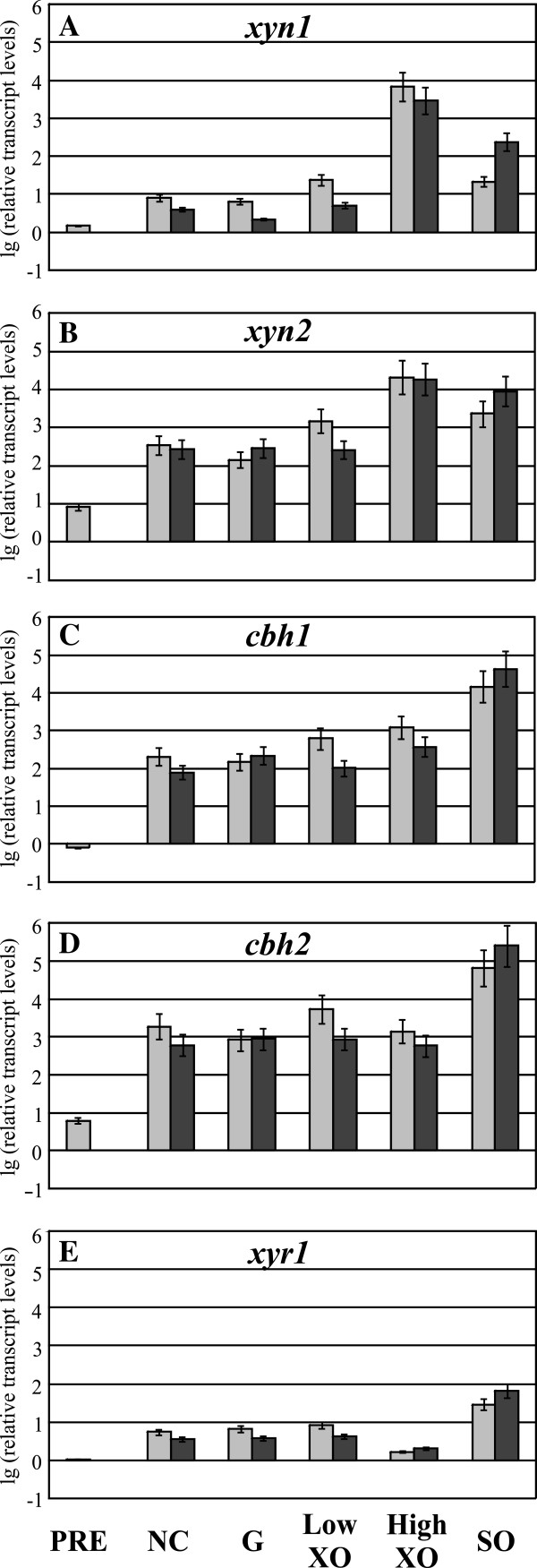
**Analysis of transcript levels of MIHCs-encoding genes and *xyr1 *in *T. reesei *Iogen-M4.** The parental strain of Iogen-M8, Iogen-M4, was precultured on glycerol and thereafter transferred to MA media without carbon source (NC) or containing 50 mM (w/v) glucose (**G**) or D-xylose (XO) at the indicated concentration, or 1.5 mM sophorose (SO) as an inducer. Samples were taken directly before transfer of mycelium (PRE), after 3 h of incubation (light grey bars), and after 6 h of incubation (dark grey bars). Transcription analyses of indicated genes (**A-E**) were performed via qPCR using *sar1* and *act* transcript levels for normalization. Results are given as relative transcript ratios in logarithmic scale (lg). The values provided in the figures are means from three biological experiments. Error bars indicate standard deviations. Transcript levels always refer to the reference sample (wild-type QM6a, NC 3 h).

The comparison of transcript levels of all MIHCs-encoding genes revealed strong differences between Iogen-M8 and Rut-C30. As two mutation/selection steps gap these two strains, we examined the intermediate strain Iogen-M4 in order to see what the first step and what the second step contribute to the observed phenotype of Iogen-M8. Therefore, we performed a carbon source replacement experiment applying the same conditions as before and analyzed the transcript levels. Like Iogen-M8, Iogen-M4 also exhibits higher transcript levels of all investigated genes under all conditions tested compared to Rut-C30 (compare Figure 3A-B to Figure 2F-J). However, the rise of transcript levels is less pronounced in Iogen-M4 than in Iogen-M8 (compare Figure 3A-E to Figure 2A-E). D-xylose at low and high concentrations induces *xyn1* and *xyn2* (compared to no carbon source) in Iogen-M4 (Figure 3A, B). The same is true for Rut-C30 (Figure 2F, G), while in Iogen-M8 the high *xyn1* and *xyn2* transcript levels remain unaffected regardless if D-xylose is applied or not (Figure 2A, B). Sophorose induces *cbh1* and *cbh2* gene expression in Iogen-M4 (Figure 3C-E) as already observed before for Rut-C30 (Figure 2H-J) and, to a lesser extent, for Iogen-M8 (Figure 2C-E). In summary, we found elevated transcript levels of all MIHCs-encoding genes already in Iogen-M4 (compared to Rut-C30), but to less pronounced extent than in Iogen-M8. Moreover, the deregulation of xylanase gene expression observed in Iogen-M8 cannot be seen in Iogen-M4.

### A824V transition in Xyr1 causes strong deregulation of xylanase expression and elevated basal cellulase expression

**Figure 4:**
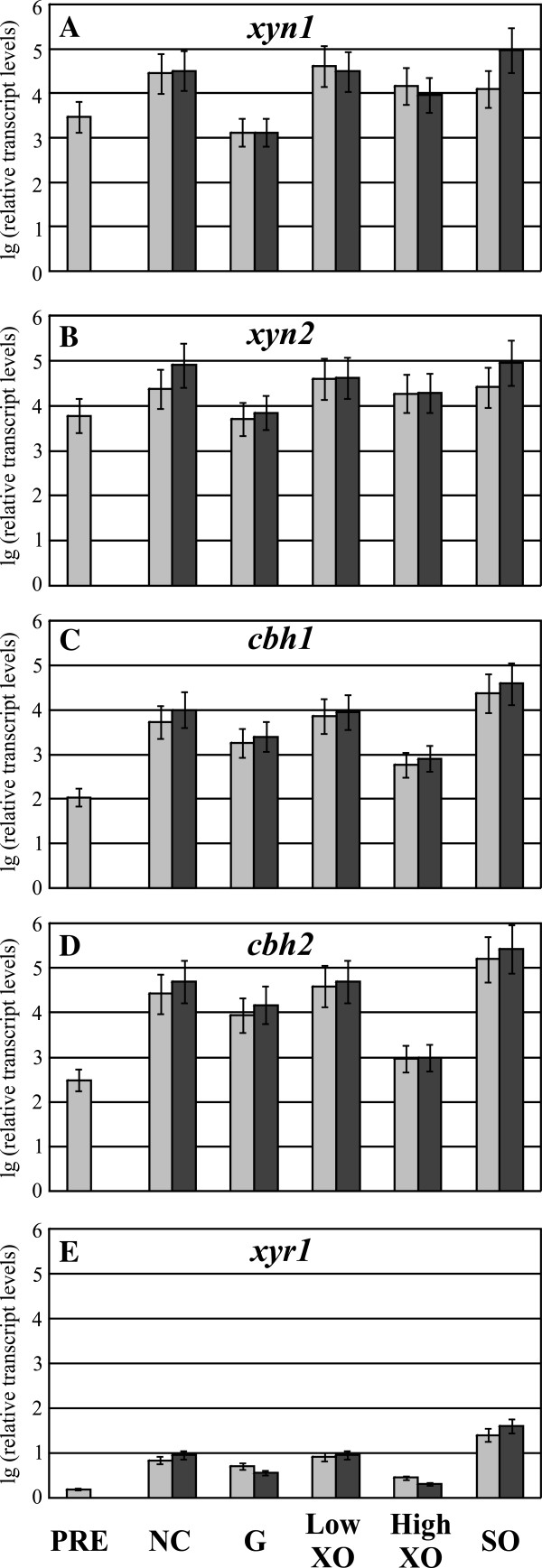
**Analysis of transcript levels of MIHCs-encoding genes and *xyr1 *in *T. reesei *Iogen-M4X.** Iogen-M4X, which is Iogen-M4 with the mutated Xyr1, was precultured on glycerol and thereafter transferred to MA media without carbon source (NC) or containing 50 mM (w/v) glucose (**G**) or D-xylose (XO) at the indicated concentration, or 1.5 mM sophorose (SO) as an inducer. Samples were taken directly before transfer of mycelium (PRE), after 3 h of incubation (light grey bars), and after 6 h of incubation (dark grey bars). Transcription analyses of indicated genes (**A-E**) were performed via qPCR using *sar1* and *act* transcript levels for normalization. Results are given as relative transcript ratios in logarithmic scale (lg). The values provided in the figures are means from three biological experiments. Error bars indicate standard deviations. Transcript levels always refer to the reference sample (wild-type QM6a, NC 3 h).

As previously stated, 17 point mutations in ORFs were identified in Iogen-M8 in comparison to Iogen-M4. We found that the A824V transition in Xyr1 leads to the elevated expression of xylanases in Iogen-M8. In order to test whether this mutation is solely responsible for all observed differences in transcript levels of Iogen-M4 and Iogen-M8, we performed transcript analysis of Iogen-M4X. This strain is isogenic to Iogen-M4 with the only exception of expressing the altered A824V (i.e. the Iogen-M8 like) Xyr1 (Figure 1A). Transcript levels of all investigated genes (*xyn1*, *xyn2*, *cbh1*, *cbh2*, *xyr1*) were nearly the same in Iogen-M4X and Iogen-M8 (compare Figure 2A-E to Figure 4A-E). Consequently, we conclude that the A824V transition in Xyr1 is responsible for the strong deregulation of xylanase expression and the additional rise in transcript levels of *xyn1* and *xyn2* observed in Iogen-M8 (higher than in Iogen-M4) as well as the high basal transcript levels of *cbh1* and *cbh2*.

In accordance with all other strains we could observe that *xyr1*, *cbh1,* and *cbh2* are inducible by sophorose in Iogen-M4X (Figure 4C-E). As a consequence of this observation, we questioned if this is due to a principle regulatory mechanism also present in the wild-type QM6a and not restricted to the strains investigated in this study so far (i.e., a consequence of mutagenesis and selection).

### Gene expression of *cbh1* and *cbh2* strictly depends on the level of *xyr1* transcription

Notably, transcript levels of *xyr1* are induced on sophorose in all strains investigated in this study so far (Figure 2E, J, Figure 3E, and Figure 4E). Moreover, the patterns of transcript levels of *cbh1* and *cbh2* strictly reflect those of *xyr1* under all conditions tested (Figure 2C-E, Figure 2H-J, Figure 3C-E, and Figure 4C-E). As mentioned, all these strains are derivatives of Rut-C30, a mutant derived from the wild-type strain QM6a (Figure 1A). We questioned whether the correlation between *xyr1* transcript levels and those of *cbh1* and *cbh2* can already be observed in the wild-type strain and performed an analogous carbon source replacement experiment using QM6a.

**Figure 5:**
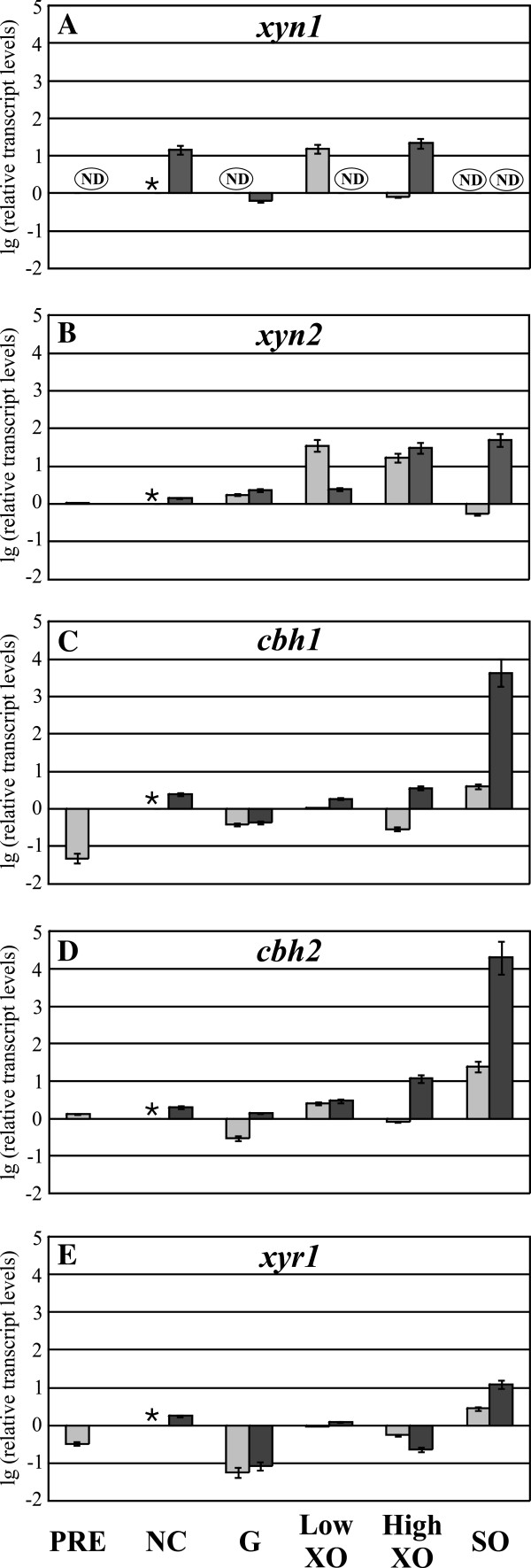
**Analysis of transcript levels of MIHCs-encoding genes and *xyr1 *in *T. reesei *QM6a.** The wild-type QM6a was precultured on glycerol and thereafter transferred to MA media without carbon source (NC) or containing 50 mM (w/v) glucose (**G**) or D-xylose (XO) at the indicated concentration, or 1.5 mM sophorose (SO) as an inducer. Samples were taken directly before transfer of mycelium (PRE), after 3 h of incubation (light grey bars), and after 6 h of incubation (dark grey bars). Transcription analyses of indicated genes (**A-E**) were performed via qPCR using *sar1* and *act* transcript levels for normalization. Results are given as relative transcript ratios in logarithmic scale (lg). The values provided in the figures are means from three biological experiments. Error bars indicate standard deviations. Transcript levels always refer to the reference sample (wild-type QM6a, NC 3 h). * indicates the reference sample, ND means not detected.

Sophorose induces *xyr1* transcription also in the wild-type (Figure 5E). In concordance with the results obtained with the other strains, *cbh1* and *cbh2* transcript levels are also elevated in QM6a on sophorose and generally reflect the carbon source/inducer-dependent pattern of *xyr1* transcript levels (Figure 5C-E). In summary, elevated levels of *xyr1* seem to correlate directly with up-regulation of *cbh1* and *cbh2* transcription in all investigated *T. reesei* strains (compare Figures 2, 3, 4 and 5C-E). In contrast, xylanase expression in all investigated strains including the wild-type strain does not follow changes in *xyr1* expression levels, i.e. Xyr1 transactivates the xylanase regulon in a concentration-independent manner (compare Figures 2, 3, 4 and 5A, B, E).

### *In silico* characterization of the *xyr1* sequence part that bears the A824V transition

According to DELTA-BLAST (http://www.ncbi.nlm.nih.gov/), Xyr1 has a so-called fungal transcription factor regulatory middle homology region (FTFRMH region) (from L359 to L904, cd12148 with an E-value of 1.77e^-11^) located next to the Gal4-like DNA-binding domain (from R93 to Y126, cd00067 with an E-value of 2.81e^-10^). The A824V mutation of Xyr1 is located within the FTFRMH region. This FTFRMH region is present in the large family of fungal zinc cluster transcription factors that contain an N-terminal GAL4-like Cys6 zinc binuclear cluster DNA-binding domain [17]. The C-terminal domain of Cep3p, a subunit of the yeast centromere-binding factor 3, is similar to the FTFRMH region (E-value 1.22e^-04^). A 3D model is available for a great part of Cep3p based on X-ray diffraction [18]. An alignment of the FTFRMH regions of Xyr1, Cep3p, and the consensus sequence matches position A824 of Xyr1 with I463 of Cep3p (Constraint-based Multiple Alignment Tool, http://www.ncbi.nlm.nih.gov/) and is shown in an additional file [see Additional file 3]. I463 of Cep3p is located in the middle of an α-helix, which reaches from M458 to I475. A graphic display is given in an additional file (see Additional file 4). Additionally, three different secondary structure predictions for the domain of Xyr1 locate A824 in the middle of an α-helix (http://www.compbio.dundee.ac.uk/, BCL::Jufo9D at http://meilerlab.org/, http://bioinf.cs.ucl.ac.uk/psipred/). Consequently, we assume that the A824V mutation in Xyr1 possibly leads to a change in secondary structure.

## Discussion

During this study we found that the expression of the two major cellulase genes *cbh1* and *cbh2* strictly follow *xyr1* transcript levels. Accordingly, Portnoy and co-workers reported that in a cellulase-overproducing strain, *xyr1* transcript levels are elevated compared to common *T. reesei* strains (as QM9414) [19]. These findings suggest that cellulase gene expression is highly dependent on the amount of Xyr1.

Otherwise, we found that the expression of the two major xylanases, *xyn1* and *xyn2,* does not strictly follow *xyr1* transcript levels. Interestingly, in *Aspergillus nidulans* a constitutive expression of *xlnR* (the *xyr1* homolog [20]) under the *gpdA* promoter led to enhanced and continuously high *xlnA*/*B* transcript formation, while *xlnD* transcript diminished after 1 h and did not follow the *xlnR* transcript level pattern anymore [21]. Altogether, we assume that regulation of xylanase gene expression is not directly dependent on the amount of Xyr1 and seems to rely on additional mechanisms.

The different Xyr1 responsiveness of cellulases and xylanases was also observed in a *T. reesei* QM9414 strain constitutively expressing *xyr1*. There, the cellulolytic regulon of Xyr1 was positively affected, while the xylanolytic regulon was negatively affected [22]. Notably, this observations are supported by the number of *in silico* identified Xyr1-binding sites in respective promoter regions. The reported Xyr1-binding site, 5’-GGC(T/A)_3_-3’ [23-26], occurs 14 times within 1 kb of the promoter region of *cbh1*, whereas it occurs only 4 times within 1 kb of the promoter region of *xyn1*. Currently ongoing *in vivo* footprinting analysis of corresponding promoters revealed that 12 and 2 of these sites are differently contacted comparing inducing and repressing conditions, respectively (unpublished observations, Gorsche, R., Lichti, J., Mach, R.L., Mach-Aigner, A.R.). Supportively, we found during this study that sophorose, which has been known for decades as a potent cellulase inducer [15], positively influences *xyr1* expression. As stated before, expression of the two major cellulase genes *cbh1* and *cbh2* strictly follow *xyr1* transcript levels. Taken together, we assume, that induction of *cbh1* and *cbh2* by sophorose is a direct result of elevated *xyr1* transcription levels. The issues discussed so far could be observed in all of the *T. reesei* strains investigated in this study, including the wild-type QM6a.

However, two outstanding phenomena could be observed in Iogen-M8: first, a strong deregulation of xylanase expression and second, a generally very high level of transcript formation of MIHCs. By investigating correspondingly manipulated strains, we found that a single point mutation in Xyr1 (A824V) is fully responsible for the deregulation of xylanase gene expression and the high basal level of *cbh1* and *cbh2* expression. A similar phenomenon was briefly described for the XlnR in *A. niger*. A V756F mutation resulted in constant xylanase activity even under repressing conditions [27]. Nonetheless, we currently cannot provide a mechanistic explanation, it is noteworthy that the A824V transition is located in a predicted α-helix within a FTFRMH region. As alanine has the lowest helix propensity (0 kcal/mol), whereas valine has a higher value (0.61 kcal/mol) [28]. The mutation in Xyr1 may result in a structural change. Notably, the previously mentioned V756 in XlnR corresponds to V821 in Xyr1 located in the same predicted α-helix as A824.

For Gal4 it was reported that glucose has a direct effect on its activity. The localisation of the glucose response domain in Gal4 was narrowed down to a central region [29], in which the FTFRMH region lies (E-value 4.57e^-50^). Albeit a functional similarity of both regions seems likely, we found that the phenotype of Iogen-M8 is not linked just to D-glucose. Consequently, we presume that the corresponding domain of Xyr1 is a more generally regulatory region.

## Conclusions

We have shown that a single point mutation in a regulatory domain of the central regulator Xyr1 has tremendous effects on expression behaviour of MIHCs in an industrially used strain of *T. reesei*. We believe that this finding is a very promising starting point for directed strain developments by e.g. transcription factor engineering. Supporting results from *A. niger* suggest that the observed phenomenon is not limited to *Trichoderma*. Therefore, we recommend manipulations of the regulatory domain of this group of Gal4-like transcription factors as a strategy for inducer-independent expression of MIHCs.

## Methods

### Fungal strains

The following *T. reesei* strains were used throughout this study: the wild-type strain QM6a (ATCC 13631), Rut-C30, which was described as a high yielding cellulase mutant of QM6a (ATCC 56765) [14], Iogen-M4, which is a spontaneous mutant of Rut-C30 [30], Iogen-M8, which is a strain obtained by UV mutation from Iogen-M4, Iogen-M4X, which is a derivate of Iogen-M4 bearing an introduced point mutation (A824V) in Xyr1, and Iogen-M8X, which is a derivate of Iogen-M8 bearing a reconstituted wild-type *xyr1*. All strains were maintained on malt extract agar or potato-dextrose-agar.

### UV mutagenesis and screening

In order to obtain Iogen-M8, conidia of Iogen-M4 from a single potato dextrose plate were suspended in 10 ml of sterile distilled water and filtered through glass wool to remove any mycelia. The conidia were diluted in water to a concentration of 10^5^ per mL and irradiated in a thin film with a germicidal lamp at a distance of 7 cm. Irradiation for 90–120 seconds was generally sufficient to give 1 - 10% survival. Diluted suspensions were plated onto selective media containing acid swollen cellulose as the primary carbon source and 4 g L^-1^ of the glucose anti-metabolite 2-deoxyglucose. Iogen-M8 was selected for its ability to grow and produce large clearing zones on this medium, indicative of hyperproduction of cellulose-degrading enzymes.

### Growth conditions

For carbon source replacement experiments, mycelia were pre-cultured in 1 L Erlenmeyer flasks on a rotary shaker (180 rpm) at 30°C for 18 h in 300 mL of Mandels-Andreotti (MA) medium [31] containing 105 mM of glycerol as the sole carbon source. A total of 10^9^ conidia per liter (final concentration) was used as the inoculum. Pre-grown mycelia were washed, and equal amounts were resuspended in MA media containing D-xylose, D-glucose, and sophorose in concentrations as stated. Mycelia were also grown in MA media without any carbon source (control). Samples were taken directly before the carbon source replacement (after harvesting the mycelia after pre-growth), after 3 hours, and after 6 hours of incubation. Samples were derived from three biological replicates and were pooled before RNA extraction.

Cultivations in a bioreactor were run in a 14 L pilot scale fermentation vessel (Model MF114 New Brunswick Scientific Co.) set up with 10 L of Initial Pilot Media. Operational parameters were: agitation at 500 rpm, air sparging at 8 standard L min^-1^, a temperature of 28°C, and pH was maintained at 4.0 - 4.5 during batch growth and pH 5.0 during enzyme production. An additional file provides a more detailed bioreactor protocol (see Additional file 5).

Growth on xylan plates was performed using MA medium containing 0.2% (w/v) azo-xylan (Megazym, Wicklow, Ireland) at 30°C.

### Determining the relative concentrations of cellulases and hemicellulases

The relative concentrations of cellulases and hemicellulase mixtures in the culture supernatants produced in bioreactors were determined by ELISA. Supernatants and purified component standards were diluted 1–100 μg mL^-1^ in phosphate-buffered saline, pH 7.2 (PBS) and incubated overnight at 4°C in microtitre plates. The plates were washed with PBS containing 0.1% Tween 20 (PBS/Tween) and incubated in PBS containing 1% BSA (PBS/BSA) for 1 h at room temperature (RT) followed by washing with PBS/Tween. Rabbit polyclonal antisera specific for CBHI, CBHII, EGI, XYNI, XYNII, and BGLI were diluted in PBS/BSA, added to separate microtitre plates and incubated for 2 h at RT. Plates were washed and incubated with a goat anti-rabbit antibody coupled to horseradish peroxidase for 1 h at RT. After washing, tetramethylbenzidine was added and incubated for 1 h at RT. The absorbance at 660 nm was measured and converted into protein concentration using the standards. The relative concentration refers to the total protein concentration of the culture supernatants.

### Isolation of chromosomal DNA and genome sequencing

After growth in 75 mL of MA medium containing 50 mM glucose at 28°C for 4 days, cultures were filtered through sterile glass fibre filters and frozen in liquid nitrogen. Biomass was ground to fine powder and resuspended in 30 mL of lysis buffer (20 mM EDTA, 10 mM Tris–HCl, pH 7.9, 1% Triton ×-100, 500 mM guanidine-HCl, 200 mM NaCl, 0.76 mg mL^-1^ Driselase® 0.4 mg mL^-1^ *T. harzianum* beta-glucanase, and 0.8 μg mL^-1^ *T. viride* chitinase C). After treatment with RNase A and RNase T1 at 20 μg mL^-1^ and 100 U mL^-1^ final concentrations (50°C, 1 h), Proteinase K was added to a concentration of 0.8 mg mL^-1^ (50°C, 1 h). Following centrifugation (20 min at 12,000 × *g*)*,* the clarified lysate was used to isolate chromosomal DNA using the Qiagen® Genomic-tip 500/G genomic DNA isolation kit (Qiagen Inc.-Canada, Ontario, CA).

Genomic DNA was sequenced using the Illumina/Solexa GAIIx sequencing technology (as distributed by Montreal Biotech Inc., Quebec, Canada) utilizing two lanes per strain (one lane of single read and one lane of paired-end reads). The raw sequences were assembled directly against publicly available sequence for strain QM6a (http://genome.jgi-psf.org/Trire2/Trire2.info.html) using DNAstar Seqman NGen® software (DNASTAR Inc., Wisconsin, USA). After assembly, a single nucleotide polymorphism calling procedure was used to identify a table of high-confidence sequence variants.

### Plasmid construction

A 2.6 kb fragment comprising the 3’-UTR of *T. reesei xyr1* was amplified from Iogen-M4 gDNA using primers FT161f and FT162r. Primer sequences are provided in Table 1. The product was used as a template for a second PCR using primers FT163f and FT164r to generate flanking sequences used in the subsequent recombination steps. Using the In-Fusion HD Cloning System (Clontech Laboratories Inc., California, USA), the second product was recombined with pNCBgl-NN that had been linearized by *Nhe*I digestion to produce the intermediate plasmid, pSC1. pNCBgl-NN contains a 3.2 kb insert comprising the promoter, coding region, and terminator of the *N. crassa pyr4* gene isolated from plasmid pFB6 [32] and a polylinker with unique *Nhe*I and *Not*I sites located at the 3‘end of the *pyr4* insert.

**Table 1:** Oligonucleotides used in this study

**Name**	**Sequence (5′ - 3′)**	**Usage**
act1f	TGAGAGCGGTGGTATCCACG	qPCR
act1r	GGTACCACCAGACATGACAATGTTG	
cbh1f	GATGATGACTACGCCAACATGCTG	
cbh1r	ACGGCACCGGGTGTGG	
cbh2f	CTATGCCGGACAGTTTGTGGTG	
cbh2r	GTCAGGCTCAATAACCAGGAGG	
taqxyn2f	GGTCCAACTCGGGCAACTTT	
taqxyn2r	CCGAGAAGTTGATGACCTTGTTC	
taqxyr1f	CCCATTCGGCGGAGGATCAG	
taqxyr1r	CGAATTCTATACAATGGGCACATGGG	
sar1fw	TGGATCGTCAACTGGTTCTACGA	
sar1rev	GCATGTGTAGCAACGTGGTCTTT	
xyn1f	CAGCTATTCGCCTTCCAACAC	
xyn1r	CAAAGTTGATGGGAGCAGAAG	
FT161f	GGAGGCCACTCAATCGTATGA	*xyr1* 3′-UTR cloning
FT162r	CGTGCCGCAATCCGGTTGTT	
FT163f	TGGGTGGTGGTATAGTCTTAAGGGAGGCCACTCAATCGTATGA	
FT164r	ACGGCCAGTGAATTCTTAATTAACGTGCCGCAATCCGGTTGTT	
FT165f	GATGTGGCAGCCGGGGAA	*xyr1* 5′-UTR and coding sequence cloning
FT166r	TTAGAGGGCCAGACCGGTTC	
FT167f	ACTCTAGATTAATTAAGATGTGGCAGCCGGGGAA	
FT168r	GCTTTCGCCACGGAGCTTTAGAGGGCCAGACCGGTTC	
FT169f	TGCCTGCAGGTCGACTCTAGATTAATTAAGATGTGGCAGCCGGGGAA	
FT170f	CGGTCTGGCCCTCTAAAGCTCCGTGGCGAAAGCCT	*cbh1* 3′-UTR cloning
FT171r	GACGAATGATGGCGGCCGCTTTCCAGGCCGCCAGCTATG	

Separately, 4.1 kb fragments were amplified from Iogen-M4 and Iogen-M8 gDNA using primers FT165f and FT166r comprising the *xyr1* 5’-UTR and the wild-type *xyr1* or *xyr1*(A824V) coding sequence, respectively. The resulting products were used as templates for a second PCR using primers FT167f and FT166r. FT167f introduces *Xba*I and *Pac*I sites at the 5’-end of the amplified products. These PCR products were used as templates for a third PCR using primers FT168r and FT169f, to generate fragments suitable for subsequent recombination steps.

A 586 bp fragment comprising the 3’-UTR of the *cbh1* gene was amplified from Iogen-M4 gDNA using primers FT170f and FT171r.

pSC1 was linearized using *Nhe*I and *Not*I and recombined with the 4.1 kb fragment containing the *xyr1* 5’-UTR and *xyr1* or *xyr1*(A824V) coding sequence and the 586 bp *cbh1* 3’-UTR fragment using the In-Fusion HD Cloning System (Clontech). This resulted in pSCxyr1-TV and pSCxyr1A824V-TV used for fungal transformation. Vector maps are provided in an additional file (see Additional file 6).

### Protoplast transformation

The protoplast transformation of *T. reesei* was performed as described in U.S. Patent No. 8,323,931. To obtain Iogen-M4X, the plasmid pSCxyr1^A824V^-TV was transformed into a uridine auxotroph of Iogen-M4 selecting for uridine prototrophy on modified (MA) medium [31]. Introduction of pSCxyr1-TV into a uridine auxotroph of Iogen-M8, followed by selection for uridine prototrophy, resulted in a mutant strain bearing the wild-type *xyr1* namely Iogen-M8X.

### RNA-extraction and reverse transcription

Harvested mycelia were homogenized in 1 mL of peqGOLD TriFast DNA/RNA/protein purification system reagent (PEQLAB Biotechnologie, Erlangen, Germany) using a FastPrep FP120 BIO101 ThermoSavant cell disrupter (Qbiogene, Carlsbad, US). RNA was isolated according to the manufacturer’s instructions, and the concentration was measured using the NanoDrop 1000 (Thermo Scientific, Waltham, US).

Synthesis of cDNA from mRNA was carried out using the RevertAid™ H Minus First Strand cDNA Synthesis Kit (Fermentas, St. Leon-Rot, Germany) according to the manufacturer’s instructions.

### Quantitative PCR analysis

Quantitative PCRs were performed in a Mastercycler® ep realplex 2.2 system (Eppendorf, Hamburg, Germany). All reactions were performed in triplicate. The amplification mixture (final volume 25 μL) contained 12.5 μL 2 × iQ SYBR Green Mix (Bio-Rad Laboratories, Hercules, USA), 100 nM forward and reverse primer and 2.5 μL cDNA (diluted 1:100). Primer sequences are provided in Table 1. Cycling conditions and control reactions were performed as described previously [33]. Data normalization using *sar1* and *act* as reference genes, and calculations were performed as published previously [33]. The transcript levels in all figures were referred to those from QM6a incubated without carbon source for 3 h; therefore, they can be compared cross-figure wisely.

## Abbreviations

CCR: Carbon catabolite repression; FTFRMH: Fungal transcription factor regulatory middle homology region; MA: Mandels Andreotti; MIHCs: Major, industrially relevant hemicellulases and cellulases; PBS: Phosphate-buffered saline; RT: Room temperature; BSA: Bovine serum albumine; Xyr1: Xylanase regulator 1.

## Competing interests

LGS, SC, and TW are current or former employees of Iogen Corporation, which has a commercial interest in the *T. reesei* strains used in this study.

## Authors’ contributions

CD carried out transcript analyses and *in silico* analyses and drafted parts of the manuscript. LGS participated in conception of the study and supervision of experiments. SC constructed strains bearing a mutated Xyr1 and carried out the characterization on the protein level. TW participated in supervision of experiments and revised the manuscript critically. RLM participated in conception of the study and revised the manuscript critically. ARMA participated in conception of the study and prepared the manuscript. All authors read and approved the final manuscript.

## Supplementary Material

Additional file 1 **Protein composition and abundance of fermentation supernatant.** The relative abundance of cellulases and hemicellulase components (CBHI, CBHII, EGI, BGLI, XYNI and XYNII) in bioreactor supernatants produced by *T. reesei* Iogen-M4, Iogen–M4X, Iogen-M8, and Iogen-M8X was determined by ELISA and is given in percent of total protein. Click here for file

Additional file 2 **Gene and aligned protein sequences of wild-type and mutated *xyr1*/Xyr1.** Intron sequences are shown in italics. Note: The *Trichoderma reesei* genome database (http://genome.jgi-psf.org/Trire2/Trire2.home.html) annotates 3 introns in the *xyr1* mRNA. cDNA sequencing revealed that the middle intron is in fact translated and therefore included in the given sequence. Positions of the mutated site are highlighted in yellow. Click here for file

Additional file 3 **Alignment of the fungal transcription factor regulatory middle homology region (FTFRMH region) of Xyr1 and Cep3p and the consensus.** Protein sequences of the FTFRMH region of Xyr1 and Cep3p and the consensus sequence were aligned with COBALT (http://www.ncbi.nlm.nih.gov/tools/cobalt/). Position 824 in Xyr1 is highlighted in red. The helix at M485 to I475 in Cep3p is highlighted in yellow. Xyr1_dom, FTFRMH region of Xyr1; Cep3p_dom, FTFRMH region of Cep3; Consensus, consensus sequence of the FTFRMH region. Click here for file

Additional file 4 **Graphical representation of 3D structure of dimerized chain A of Cep3p.** Protein 3D structure of chain A of Cep3p (PDB: 2VEQ_A) visualized with Cn3D 4.3. Pictures show a dimer from 3 angles, respectively. The helix at M458 to I475 is highlighted in yellow in both dimers. Click here for file

Additional file 5 Detailed protocol for cultivation in a bioreactor. Click here for file

Additional file 6 **Vector maps of pSCxyr1-TV and pSCxyr1A824V-TV.** Maps of the vectors pSCxyr1-TV and pSCxyr1A824V-TV used to generate Iogen M8X and Iogen-M4X, respectively. Vectors were digested with *Pac*I prior to transformation of strains Iogen-M8 and Iogen-M4. Click here for file
