# Supplementary figures and images for: Mutation of the Xylanase regulator 1 causes a glucose blind hydrolase expressing phenotype in industrially used Trichoderma strains
Source: Biotechnol Biofuels. 2013 May 2;6:62. doi: 10.1186/1754-6834-6-62 (PMC3654998; doi:10.1186/1754-6834-6-62)

Additional file 1 - Protein composition and abundance of fermentation supernatant

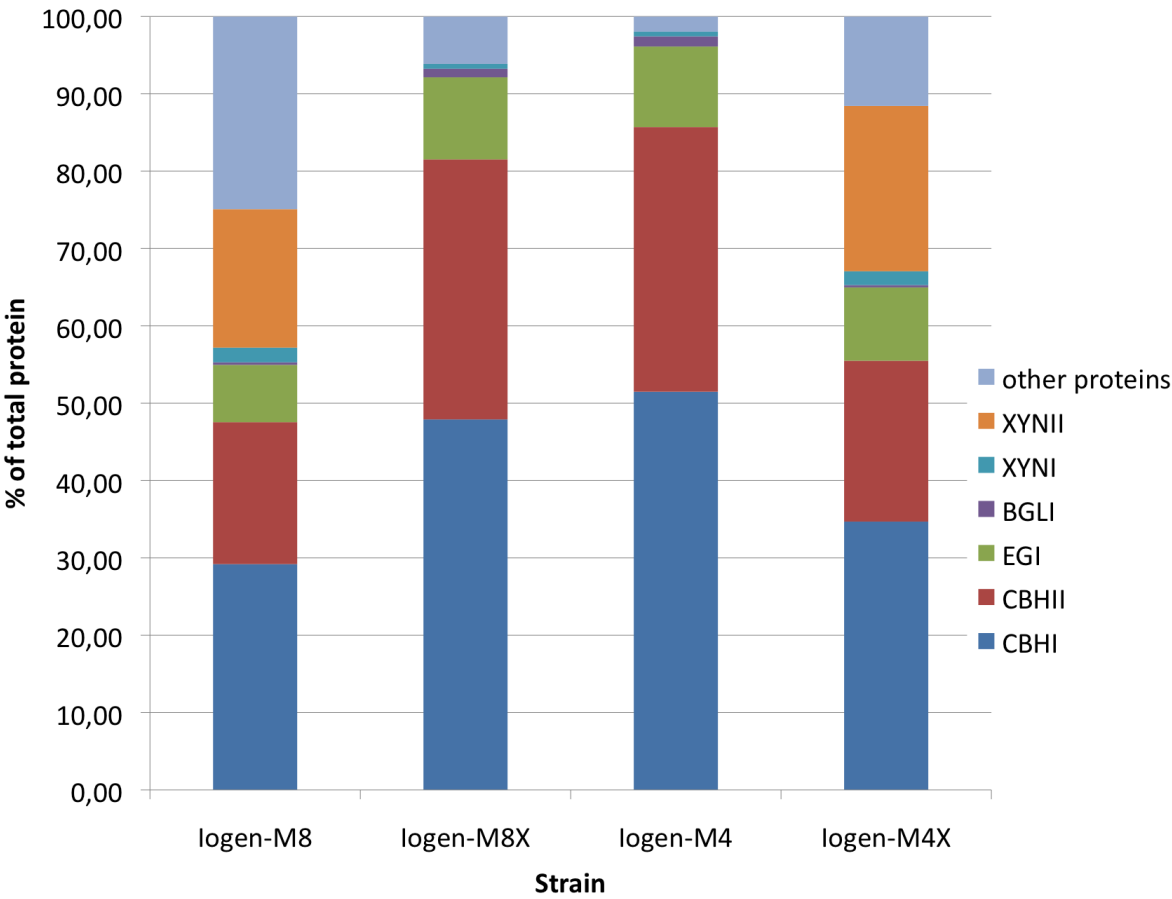

Supplement: Additional file 1 — Protein composition and abundance of fermentation supernatant. The relative abundance of cellulases and hemicellulase components (CBHI, CBHII, EGI, BGLI, XYNI and XYNII) in bioreactor supernatants produced by T. reesei Iogen-M4, Iogen–M4X, Iogen-M8, and Iogen-M8X was determined by ELISA and is given in percent of total protein. [file 1754-6834-6-62-S1.pdf]

**Additional file 4 - Graphical representation of 3D structure of dimerized chain A of Cep3p**

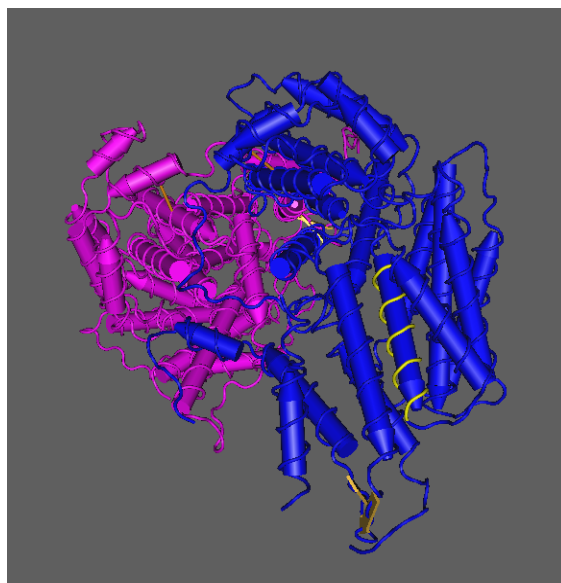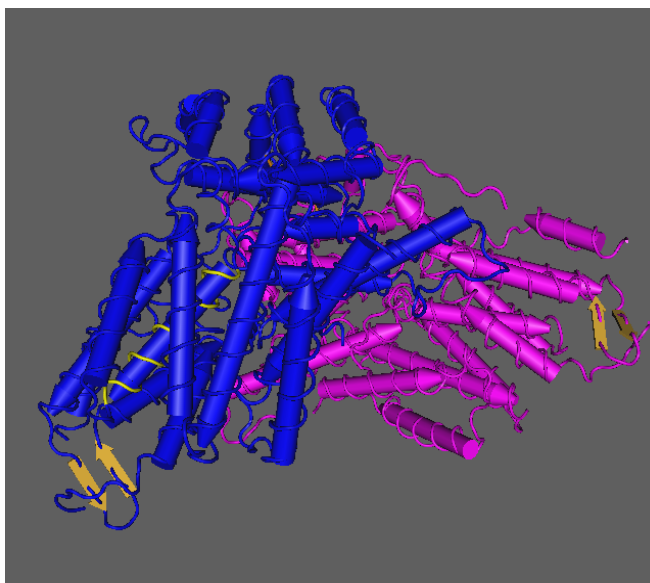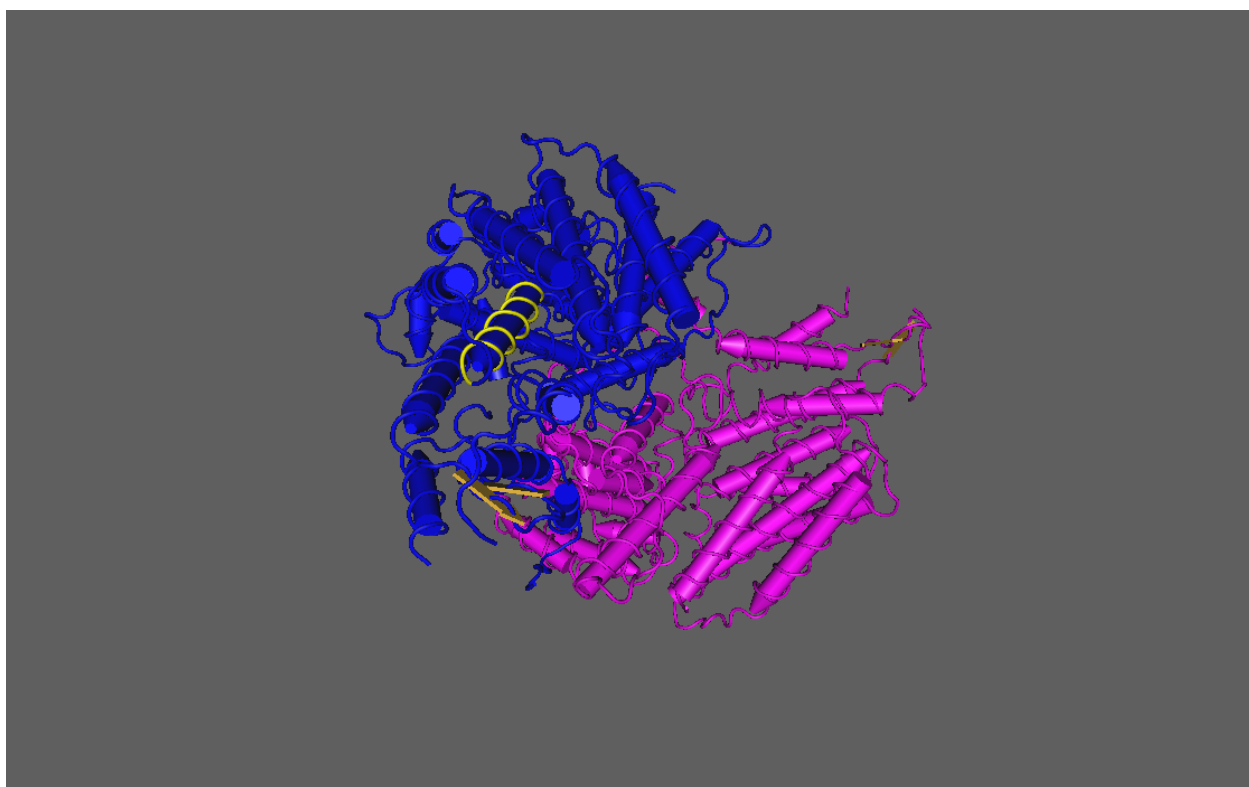

Supplement: Additional file 4 — Graphical representation of 3D structure of dimerized chain A of Cep3p. Protein 3D structure of chain A of Cep3p (PDB: 2VEQ_A) visualized with Cn3D 4.3. Pictures show a dimer from 3 angles, respectively. The helix at M458 to I475 is highlighted in yellow in both dimers. [file 1754-6834-6-62-S4.pdf]

Additional file 6 - Vector maps of pSCxyr1-TV and pSCxyr1A824V-TV

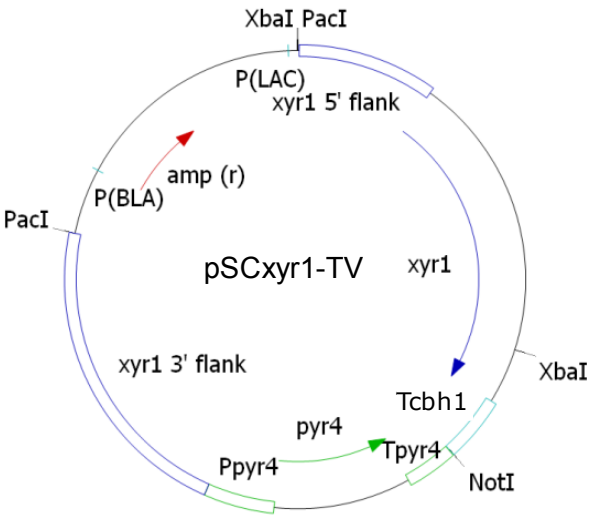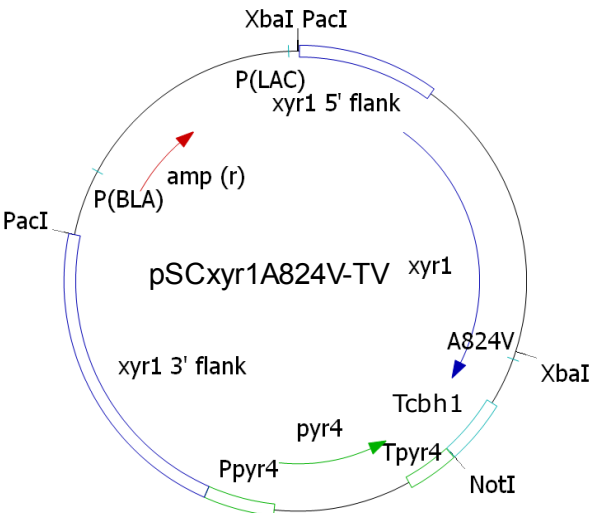

Supplement: Additional file 6 — Vector maps of pSCxyr1-TV and pSCxyr1A824V-TV. Maps of the vectors pSCxyr1-TV and pSCxyr1A824V-TV used to generate Iogen M8X and Iogen-M4X, respectively. Vectors were digested with PacI prior to transformation of strains Iogen-M8 and Iogen-M4. [file 1754-6834-6-62-S6.pdf]
