# Supplementary material for: Mutation of the Xylanase regulator 1 causes a glucose blind hydrolase expressing phenotype in industrially used Trichoderma strains
Source: Biotechnol Biofuels. 2013 May 2;6:62. doi: 10.1186/1754-6834-6-62 (PMC3654998; doi:10.1186/1754-6834-6-62)
Supplement: Additional file 2 — Gene and aligned protein sequences of wild-type and mutated xyr1/Xyr1. Intron sequences are shown in italics. Note: The Trichoderma reesei genome database (http://genome.jgi-psf.org/Trire2/Trire2.home.html) annotates 3 introns in the xyr1 mRNA. cDNA sequencing revealed that the middle intron is in fact translated and therefore included in the given sequence. Positions of the mutated site are highlighted in yellow. [file 1754-6834-6-62-S2.pdf]

Additional file 2

(A) Gene and aligned protein sequence of wild-type *xyr1*/Xyr1

```
   1  ATGTTGTCCAATCCTCTCCGTCGCTATTCTGCCTACCCCGACATCTCCTCGGCGTCATTTGACCCGAACTACCATGGCTC
   1   M  L  S  N  P  L  R  R  Y  S  A  Y  P  D  I  S  S  A  S  F  D  P  N  Y  H  G  S
  81  ACAGTCGCATCTCCACTCGATCAACGTCAACACATTCGGCAACAGCCACCCCTATCCCATGCAGCACCTCGCACAGCATG
  27    Q  S  H  L  H  S  I  N  V  N  T  F  G  N  S  H  P  Y  P  M  Q  H  L  A  Q  H
 161  CGGAGCTTTCGAGTTCACGCATGATAAGGGCCAGTCCGGTGCAGCCAAAGCAGCGCCAGGGCTCTCTTATTGCTGCCAGG
  54  A  E  L  S  S  S  R  M  I  R  A  S  P  V  Q  P  K  Q  R  Q  G  S  L  I  A  A  R
 241  AAGAATTCAACGGGTACTGCTGGGCCCATTCGGCGGAGGATCAGTCGCGCTTGTGACCAGTGCAACCAGCTTCGTACCAA
  81   K  N  S  T  G  T  A  G  P  I  R  R  R  I  S  R  A  C  D  Q  C  N  Q  L  R  T  K
 321  GTGCGATGGCTTACACCCATGTGCCCATTGTATAG gtatgtccctttttcctctacacagtgatgctgcgctcaagcacat
 107    C  D  G  L  H  P  C  A  H  C  I
 401  gtactgatcgatcttgtttag AATTCGGCCTTGGATGCGAATATGTCCGAGAGAGAAAGAAGCGTGGCAAAGCTTCGCGC
 119                        E  F  G  L  G  C  E  Y  V  R  E  R  K  K  R  G  K  A  S  R
 481  AAGGATATTGCTGCCCAGCAAGCCGCGGCGGCTGCAGCACAACACTCCGGCCAGGTCCAGGATGGTCCAGAGGATCAACA
 139   K  D  I  A  A  Q  Q  A  A  A  A  A  A  Q  H  S  G  Q  V  Q  D  G  P  E  D  Q  H
 561  TCGCAAACTCTCACGCCAGCAAAGCGAATCTTCGCGTGGCAGCGCTGAGCTTGCCCAGCCTGCCCACGACCCGCCTCATG
 165    R  K  L  S  R  Q  Q  S  E  S  S  R  G  S  A  E  L  A  Q  P  A  H  D  P  P  H
 641  GCCACATTGAGGGCTCTGTCAGCTCCTTCAGCGACAATGGCCTTTCCCAGCATGCTGCCATGGGCGGCATGGATGGCCTG
 192  G  H  I  E  G  S  V  S  S  F  S  D  N  G  L  S  Q  H  A  A  M  G  G  M  D  G  L
 721  GAAGATCACCATGGCCACGTCGGAGTTGATCCTGCCCTGGGCCGAACTCAGCTGGAAGCGTCATCAGCAATGGGCCTGGG
 219   E  D  H  H  G  H  V  G  V  D  P  A  L  G  R  T  Q  L  E  A  S  S  A  M  G  L  G
 801  CGCATACGGTGAAGTCCACCCCGGCTATGAGAGCCCCGGCATGAATGGCCATGTGATGGTGCCCCCGTCGTATGGCGCGC
 245    A  Y  G  E  V  H  P  G  Y  E  S  P  G  M  N  G  H  V  M  V  P  P  S  Y  G  A
 881  AGACCACCATGGCCGGGTATTCCGGTATCTCGTATGCTGCGCAAGCCCCGAGTCCGGCTACGTATAGCAGCGACGGTAAC
 272  Q  T  T  M  A  G  Y  S  G  I  S  Y  A  A  Q  A  P  S  P  A  T  Y  S  S  D  G  N
 961  TTTCGACTCACCGGTCACATCCATGATTACCCGCTGGCAAATGGGAGCTCGCCCTCATGGGGAGTCTCGCTGGCCTCGCC
 299   F  R  L  T  G  H  I  H  D  Y  P  L  A  N  G  S  S  P  S  W  G  V  S  L  A  S  P
1041  TTCGAACCAGTTCCAGCTTCAGCTCTCGCAGCCCATCTTCAAGCAAAGCGATTTGCGATATCCTGTGCTTGAGCCTCTGC
 326    S  N  Q  F  Q  L  Q  L  S  Q  P  I  F  K  Q  S  D  L  R  Y  P  V  L  E  P  L
1121  TGCCTCACCTGGGAAACATCCTCCCCGTGTCTTTGGCGTGCGATCTGATTGACCTGTACTTCTCCTCGTCTTCATCAGCA
 352  L  P  H  L  G  N  I  L  P  V  S  L  A  C  D  L  I  D  L  Y  F  S  S  S  S  S  A
1201  CAGATGCACCCAATGTCCCCATACGTTCTGGGCTTCGTCTTCCGGAAGCGCTCCTTCTTGCACCCCACGAACCCACGAAG
 379   Q  M  H  P  M  S  P  Y  V  L  G  F  V  F  R  K  R  S  F  L  H  P  T  N  P  R  R
1281  GTGCCAGCCCGCGCTGCTTGCGAGCATGCTGTGGGTGGCGGCACAGACTAGCGAAGCGTCCTTCTTGACGAGCCTGCCGT
 405    C  Q  P  A  L  L  A  S  M  L  W  V  A  A  Q  T  S  E  A  S  F  L  T  S  L  P
1361  CGGCGAGGAGCAAGGTCTGCCAGAAGCTGCTCGAGCTGACCGTTGGGCTTCTTCAGCCCCTGATCCACACCGGCACCAAC
 432  S  A  R  S  K  V  C  Q  K  L  L  E  L  T  V  G  L  L  Q  P  L  I  H  T  G  T  N
1441  AGCCCGTCTCCCAAGACTAGCCCCGTCGTCGGTGCTGCTGCCCTGGGAGTTCTTGGGGTGGCCATGCCGGGCTCGCTGAA
 469   S  P  S  P  K  T  S  P  V  V  G  A  A  A  L  G  V  L  G  V  A  M  P  G  S  L  N
1521  CATGGATTCACTGGCCGGCGAAACGGGTGCTTTTGGGGCCATAGGGAGCCTTGACGACGTCATCACCTATGTGCACCTCG
 485    M  D  S  L  A  G  E  T  G  A  F  G  A  I  G  S  L  D  D  V  I  T  Y  V  H  L
1601  CCACGGTCGTCTCGGCCAGCGAGTACAAGGGCGCCAGCCTGCGGTGGTGGGGTGCGGCATGGTCTCTCGCCAGAGAGCTC
 512  A  T  V  V  S  A  S  E  Y  K  G  A  S  L  R  W  W  G  A  A  W  S  L  A  R  E  L
1681  AAGCTTGGCCGTGAGCTGCCGCCTGGCAATCCACCTGCCAACCAGGAGGACGGCGAGGGCCTTAGCGAAGACGTGGATGA
 539   K  L  G  R  E  L  P  P  G  N  P  P  A  N  Q  E  D  G  E  G  L  S  E  D  V  D  E
1761  GCACGACTTGAACAGAAACAACACTCGCTTCGTGACGGAAGAGGAGCGCGAAGAGCGACGGCGAGCATGGTGGCTCGTTT
 565    H  D  L  N  R  N  N  T  R  F  V  T  E  E  E  R  E  E  R  R  R  A  W  W  L  V
1841  ACATCGTCGACAGGCACCTGGCGCTCTGCTACAACCGCCCCTTGTTTCTTCTGGACAGCGAGTGCAGCGACTTGTACCAC
 592  Y  I  V  D  R  H  L  A  L  C  Y  N  R  P  L  F  L  L  D  S  E  C  S  D  L  Y  H
1921  CCGATGGACGACATCAAGTGGCAGGCAGGCAAATTTCGCAGCCACGATGCAGGGAACTCCAGCATCAACATCGATAGCTC
 619   P  M  D  D  I  K  W  Q  A  G  K  F  R  S  H  D  A  G  N  S  S  I  N  I  D  S  S
2001  CATGACGGACGAGTTTGGCGATAGTCCCCGGGCGGCTCGCGGCGCACACTACGAGTGCCGCGGTCGTAGCATTTTTGGCT
 645    M  T  D  E  F  G  D  S  P  R  A  A  R  G  A  H  Y  E  C  R  G  R  S  I  F  G
2081  ACTTCTTGTCCTTGATGACAATCCTGGGCGAGATTGTCGATGTCCACCATGCTAAAAGCCACCCCCGGTTCGGCGTTGGA
 672  Y  F  L  S  L  M  T  I  L  G  E  I  V  D  V  H  H  A  K  S  H  P  R  F  G  V  G
2161  TTCCGCTCCGCGCGGGATTGGGACGAGCAGGTTGCTGAAATCACCCGACACCTGGACATGTATGAGGAGAGCCTCAAGAG
 699   F  R  S  A  R  D  W  D  E  Q  V  A  E  I  T  R  H  L  D  M  Y  E  E  S  L  K  R
2241  GTTCGTGGCCAAGCATCTGCCATTGTCCTCAAAGGACAAGGAGCAGCATGAGATGCACGACAGTGGAGCGGTAACAGACA
 725    F  V  A  K  H  L  P  L  S  S  K  D  K  E  Q  H  E  M  H  D  S  G  A  V  T  D
2321  TGCAATCTCCACTCTCGGTGCGGACCAACGCGTCCAGCCGCATGACGGAGAGCGAGATCCAGGCCAGCATCGTGGTGGCT
 752  M  Q  S  P  L  S  V  R  T  N  A  S  S  R  M  T  E  S  E  I  Q  A  S  I  V  V  A
2401  TACAGCACCCATGTGATGCATGTCCTCCACATCCTCCTTGCGGATAAGTGGGATCCCATCAACCTTCTAGACGACGACGA
 779   Y  S  T  H  V  M  H  V  L  H  I  L  L  A  D  K  W  D  P  I  N  L  L  D  D  D  D
2481  CTTGTGGATCTCGTCGGAAGGATTCGTGACGGCGACGAGCCACGCGGTATCGGCTGCCGAAGCTATTAGCCAGATTCTCG
 805    L  W  I  S  S  E  G  F  V  T  A  T  S  H  A  V  S  A  A  E  A  I  S  Q  I  L
2561  AGTTTGACCCTGGCCTGGAGTTTATGCCATTCTTCTACGGCGTCTATCTCCTGCAGGGTTCCTTCCTCCTCCTGCTCATC
 832  E  F  D  P  G  L  E  F  M  P  F  F  Y  G  V  Y  L  L  Q  G  S  F  L  L  L  L  I
2641  GCCGACAAGCTGCAGGCCGAAGCGTCTCCAAGCGTCATCAAGGCTTGCGAGACCATTGTTAGGGCACACGAAGCTTGCGT
 859   A  D  K  L  Q  A  E  A  S  P  S  V  I  K  A  C  E  T  I  V  R  A  H  E  A  C  V
2721  TGTGACGCTGAGCACAGAGTATCAG gtaagccctatcagttcaaacgtctatcttgctgtgaatcaaagactgacttgga
 885    V  T  L  S  T  E  Y  Q
2801  catcag CGCAACTTTAGCAAGGTTATGCGAAGCGCGCTGGCTCTGATTCGGGGCCGTGTGCCGGAAGATTTAGCTGAGCA
 894          R  N  F  S  K  V  M  R  S  A  L  A  L  I  R  G  R  V  P  E  D  L  A  E  Q
2881  GCAGCAGCGACGACGCGAGCTTCTTGCACTATACCGATGGACTGGTAACGGAACCGGTCTGGCCCTCTAA
 918    Q  Q  R  R  R  E  L  L  A  L  Y  R  W  T  G  N  G  T  G  L  A  L  -
```

Additional file 2 (continued)

(B) Gene and aligned protein sequence of mutated *xyr1*/Xyr1

```
   1  ATGTTGTCCAATCCTCTCCGTCGCTATTCTGCCTACCCCGACATCTCCTCGGCGTCATTTGACCCGAACTACCATGGCTC
   1   M  L  S  N  P  L  R  R  Y  S  A  Y  P  D  I  S  S  A  S  F  D  P  N  Y  H  G  S
  81  ACAGTCGCATCTCCACTCGATCAACGTCAACACATTCGGCAACAGCCACCCCTATCCCATGCAGCACCTCGCACAGCATG
  27    Q  S  H  L  H  S  I  N  V  N  T  F  G  N  S  H  P  Y  P  M  Q  H  L  A  Q  H
 161  CGGAGCTTTCGAGTTCACGCATGATAAGGGCCAGTCCGGTGCAGCCAAAGCAGCGCCAGGGCTCTCTTATTGCTGCCAGG
  54  A  E  L  S  S  S  R  M  I  R  A  S  P  V  Q  P  K  Q  R  Q  G  S  L  I  A  A  R
 241  AAGAATTCAACGGGTACTGCTGGGCCCATTCGGCGGAGGATCAGTCGCGCTTGTGACCAGTGCAACCAGCTTCGTACCAA
  81   K  N  S  T  G  T  A  G  P  I  R  R  R  I  S  R  A  C  D  Q  C  N  Q  L  R  T  K
 321  GTGCGATGGCTTACACCCATGTGCCCATTGTATAG gtatgtccctttttcctctacacagtgatgctgcgctcaagcacat
 107    C  D  G  L  H  P  C  A  H  C  I
 401  gtactgatcgatcttgtttag AATTCGGCCTTGGATGCGAATATGTCCGAGAGAGAAAGAAGCGTGGCAAAGCTTCGCGC
 119                         E  F  G  L  G  C  E  Y  V  R  E  R  K  K  R  G  K  A  S  R
 481  AAGGATATTGCTGCCCAGCAAGCCGCGGCGGCTGCAGCACAACACTCCGGCCAGGTCCAGGATGGTCCAGAGGATCAACA
 139   K  D  I  A  A  Q  Q  A  A  A  A  A  A  Q  H  S  G  Q  V  Q  D  G  P  E  D  Q  H
 561  TCGCAAACTCTCACGCCAGCAAAGCGAATCTTCGCGTGGCAGCGCTGAGCTTGCCCAGCCTGCCCACGACCCGCCTCATG
 165    R  K  L  S  R  Q  Q  S  E  S  S  R  G  S  A  E  L  A  Q  P  A  H  D  P  P  H
 641  GCCACATTGAGGGCTCTGTCAGCTCCTTCAGCGACAATGGCCTTTCCCAGCATGCTGCCATGGGCGGCATGGATGGCCTG
 192  G  H  I  E  G  S  V  S  S  F  S  D  N  G  L  S  Q  H  A  A  M  G  G  M  D  G  L
 721  GAAGATCACCATGGCCACGTCGGAGTTGATCCTGCCCTGGGCCGAACTCAGCTGGAAGCGTCATCAGCAATGGGCCTGGG
 219   E  D  H  H  G  H  V  G  V  D  P  A  L  G  R  T  Q  L  E  A  S  S  A  M  G  L  G
 801  CGCATACGGTGAAGTCCACCCCGGCTATGAGAGCCCCGGCATGAATGGCCATGTGATGGTGCCCCCGTCGTATGGCGCGC
 245    A  Y  G  E  V  H  P  G  Y  E  S  P  G  M  N  G  H  V  M  V  P  P  S  Y  G  A
 881  AGACCACCATGGCCGGGTATTCCGGTATCTCGTATGCTGCGCAAGCCCCGAGTCCGGCTACGTATAGCAGCGACGGTAAC
 272  Q  T  T  M  A  G  Y  S  G  I  S  Y  A  A  Q  A  P  S  P  A  T  Y  S  S  D  G  N
 961  TTTCGACTCACCGGTCACATCCATGATTACCCGCTGGCAAATGGGAGCTCGCCCTCATGGGGAGTCTCGCTGGCCTCGCC
 299   F  R  L  T  G  H  I  H  D  Y  P  L  A  N  G  S  S  P  S  W  G  V  S  L  A  S  P
1041  TTCGAACCAGTTCCAGCTTCAGCTCTCGCAGCCCATCTTCAAGCAAAGCGATTTGCGATATCCTGTGCTTGAGCCTCTGC
 326    S  N  Q  F  Q  L  Q  L  S  Q  P  I  F  K  Q  S  D  L  R  Y  P  V  L  E  P  L
1121  TGCCTCACCTGGGAAACATCCTCCCCGTGTCTTTGGCGTGCGATCTGATTGACCTGTACTTCTCCTCGTCTTCATCAGCA
 352  L  P  H  L  G  N  I  L  P  V  S  L  A  C  D  L  I  D  L  Y  F  S  S  S  S  S  A
1201  CAGATGCACCCAATGTCCCCATACGTTCTGGGCTTCGTCTTCCGGAAGCGCTCCTTCTTGCACCCCACGAACCCACGAAG
 379   Q  M  H  P  M  S  P  Y  V  L  G  F  V  F  R  K  R  S  F  L  H  P  T  N  P  R  R
1281  GTGCCAGCCCGCGCTGCTTGCGAGCATGCTGTGGGTGGCGGCACAGACTAGCGAAGCGTCCTTCTTGACGAGCCTGCCGT
 405    C  Q  P  A  L  L  A  S  M  L  W  V  A  A  Q  T  S  E  A  S  F  L  T  S  L  P
1361  CGGCGAGGAGCAAGGTCTGCCAGAAGCTGCTCGAGCTGACCGTTGGGCTTCTTCAGCCCCTGATCCACACCGGCACCAAC
 432  S  A  R  S  K  V  C  Q  K  L  L  E  L  T  V  G  L  L  Q  P  L  I  H  T  G  T  N
1441  AGCCCGTCTCCCAAGACTAGCCCCGTCGTCGGTGCTGCTGCCCTGGGAGTTCTTGGGGTGGCCATGCCGGGCTCGCTGAA
 469   S  P  S  P  K  T  S  P  V  V  G  A  A  A  L  G  V  L  G  V  A  M  P  G  S  L  N
1521  CATGGATTCACTGGCCGGCGAAACGGGTGCTTTTGGGGCCATAGGGAGCCTTGACGACGTCATCACCTATGTGCACCTCG
 485    M  D  S  L  A  G  E  T  G  A  F  G  A  I  G  S  L  D  D  V  I  T  Y  V  H  L
1601  CCACGGTCGTCTCGGCCAGCGAGTACAAGGGCGCCAGCCTGCGGTGGTGGGGTGCGGCATGGTCTCTCGCCAGAGAGCTC
 512  A  T  V  V  S  A  S  E  Y  K  G  A  S  L  R  W  W  G  A  A  W  S  L  A  R  E  L
1681  AAGCTTGGCCGTGAGCTGCCGCCTGGCAATCCACCTGCCAACCAGGAGGACGGCGAGGGCCTTAGCGAAGACGTGGATGA
 539   K  L  G  R  E  L  P  P  G  N  P  P  A  N  Q  E  D  G  E  G  L  S  E  D  V  D  E
1761  GCACGACTTGAACAGAAACAACACTCGCTTCGTGACGGAAGAGGAGCGCGAAGAGCGACGGCGAGCATGGTGGCTCGTTT
 565    H  D  L  N  R  N  N  T  R  F  V  T  E  E  E  R  E  E  R  R  R  A  W  W  L  V
1841  ACATCGTCGACAGGCACCTGGCGCTCTGCTACAACCGCCCCTTGTTTCTTCTGGACAGCGAGTGCAGCGACTTGTACCAC
 592  Y  I  V  D  R  H  L  A  L  C  Y  N  R  P  L  F  L  L  D  S  E  C  S  D  L  Y  H
1921  CCGATGGACGACATCAAGTGGCAGGCAGGCAAATTTCGCAGCCACGATGCAGGGAACTCCAGCATCAACATCGATAGCTC
 619   P  M  D  D  I  K  W  Q  A  G  K  F  R  S  H  D  A  G  N  S  S  I  N  I  D  S  S
2001  CATGACGGACGAGTTTGGCGATAGTCCCCGGGCGGCTCGCGGCGCACACTACGAGTGCCGCGGTCGTAGCATTTTTGGCT
 645    M  T  D  E  F  G  D  S  P  R  A  A  R  G  A  H  Y  E  C  R  G  R  S  I  F  G
2081  ACTTCTTGTCCTTGATGACAATCCTGGGCGAGATTGTCGATGTCCACCATGCTAAAAGCCACCCCCGGTTCGGCGTTGGA
 672  Y  F  L  S  L  M  T  I  L  G  E  I  V  D  V  H  H  A  K  S  H  P  R  F  G  V  G
2161  TTCCGCTCCGCGCGGGATTGGGACGAGCAGGTTGCTGAAATCACCCGACACCTGGACATGTATGAGGAGAGCCTCAAGAG
 699   F  R  S  A  R  D  W  D  E  Q  V  A  E  I  T  R  H  L  D  M  Y  E  E  S  L  K  R
2241  GTTCGTGGCCAAGCATCTGCCATTGTCCTCAAAGGACAAGGAGCAGCATGAGATGCACGACAGTGGAGCGGTAACAGACA
 725    F  V  A  K  H  L  P  L  S  S  K  D  K  E  Q  H  E  M  H  D  S  G  A  V  T  D
2321  TGCAATCTCCACTCTCGGTGCGGACCAACGCGTCCAGCCGCATGACGGAGAGCGAGATCCAGGCCAGCATCGTGGTGGCT
 752  M  Q  S  P  L  S  V  R  T  N  A  S  S  R  M  T  E  S  E  I  Q  A  S  I  V  V  A
2401  TACAGCACCCATGTGATGCATGTCCTCCACATCCTCCTTGCGGATAAGTGGGATCCCATCAACCTTCTAGACGACGACGA
 779   Y  S  T  H  V  M  H  V  L  H  I  L  L  A  D  K  W  D  P  I  N  L  L  D  D  D  D
2481  CTTGTGGATCTCGTCGGAAGGATTCGTGACGGCGACGAGCCACGCGGTATCGGCTGTCGAAGCTATTAGCCAGATTCTCG
 805    L  W  I  S  S  E  G  F  V  T  A  T  S  H  A  V  S  A  V  E  A  I  S  Q  I  L
2561  AGTTTGACCCTGGCCTGGAGTTTATGCCATTCTTCTACGGCGTCTATCTCCTGCAGGGTTCCTTCCTCCTCCTGCTCATC
 832  E  F  D  P  G  L  E  F  M  P  F  F  Y  G  V  Y  L  L  Q  G  S  F  L  L  L  L  I
2641  GCCGACAAGCTGCAGGCCGAAGCGTCTCCAAGCGTCATCAAGGCTTGCGAGACCATTGTTAGGGCACACGAAGCTTGCGT
 859   A  D  K  L  Q  A  E  A  S  P  S  V  I  K  A  C  E  T  I  V  R  A  H  E  A  C  V
2721  TGTGACGCTGAGCACAGAGTATCAG gtaagccctatcagttcaaacgtctatcttgctgtgaatcaaagactgacttgga
 885    V  T  L  S  T  E  Y  Q
2801  catcag CGCAACTTTAGCAAGGTTATGCGAAGCGCGCTGGCTCTGATTCGGGGCCGTGTGCCGGAAGATTTAGCTGAGCA
 894          R  N  F  S  K  V  M  R  S  A  L  A  L  I  R  G  R  V  P  E  D  L  A  E  Q
2881  GCAGCAGCGACGACGCGAGCTTCTTGCACTATACCGATGGACTGGTAACGGAACCGGTCTGGCCCTCTAA
 918    Q  Q  R  R  R  E  L  L  A  L  Y  R  W  T  G  N  G  T  G  L  A  L  -
```
