# Supplementary material for: Mutation of the Xylanase regulator 1 causes a glucose blind hydrolase expressing phenotype in industrially used Trichoderma strains
Source: Biotechnol Biofuels. 2013 May 2;6:62. doi: 10.1186/1754-6834-6-62 (PMC3654998; doi:10.1186/1754-6834-6-62)
Supplement: Additional file 3 — Alignment of the fungal transcription factor regulatory middle homology region (FTFRMH region) of Xyr1 and Cep3p and the consensus. Protein sequences of the FTFRMH region of Xyr1 and Cep3p and the consensus sequence were aligned with COBALT (http://www.ncbi.nlm.nih.gov/tools/cobalt/). Position 824 in Xyr1 is highlighted in red. The helix at M485 to I475 in Cep3p is highlighted in yellow. Xyr1_dom, FTFRMH region of Xyr1; Cep3p_dom, FTFRMH region of Cep3; Consensus, consensus sequence of the FTFRMH region. [file 1754-6834-6-62-S3.pdf]

Additional file 3 - Alignment of the fungal transcription factor regulatory middle homology region (FTFRMH region) of Xyr1 and Cep3p and the consensus

```
Xyr1_dom     359  LPVSLACDLIDLYFSSSSSAQMHPMSPYVLGFVFRKRSFLHPTNPRRCQPALLASMLWVA
Cep3p_dom         ------------------------------------------------------------
Consensus      1  PPREVADRLLDLYFEN-----VHPLFPILHRPTFLRDLESLYSDPSSLSPASLALLLAVL

Xyr1_dom     419  AQTSEASFLTSLPSA------RSKVCQKLLELTVGLLQPLIHTGTNSPSPKTSPVVGAAA
Cep3p_dom    108  -----------------------------KDQSFQLMNFAMEN------------LGALY
Consensus     56  ALAA-----LSLPDSELRGESRRSLAERYYEAARQLLDLALFL---PPSLETLQALLLLA
                                                : :  *::  :              :

Xyr1_dom     473  LGVLGVAMPGSLNMDSLAGETGAFGAIGSLDDVITYVHLATVVSASEYKGASLRWWGAAW
Cep3p_dom    127  FGSIGDISELYLRVEQYWDRRADKNH--SVDGKYWDALIWSVFTMCIY----YMPVEKLA
Consensus    108  LYLLGTGDPSS---------------------------------------AWLLLGLAI
                  :  :*

Xyr1_dom     533  SLARELKLGRELPPGNPPANQEDGEGLSEDVDEHDLNRNNTRFVTEEEREERRRAWWLVY
Cep3p_dom    181  EIFSVYPLHEYL-GSNKRLNWEDGMQLVMCQNFARCSLFQLKQCDFMAHPDIRLV--QAY
Consensus    128  RLAQSLGLHRD--PSSLP-------GLSP-----------------FERELRRRLWWSLY
                   :     * .    ..          *                     :   *      *

Xyr1_dom     593  IVDRHLALCYNRPLFLLDSECSDLYHPMDDIKWQAGKFRSHDARNSSINIDSSMT-----
Cep3p_dom    238  LILATTTFPYDEPL-LANSLLTQCIHTFKN--FHVDDFRPL-LNDDPVESIAKVTLGRIF
Consensus    162  ILDRLLSLSLGRPPSISDEDID-------------------------VPLPSNED-----
                  ::    ::  ..*  : :.                            :   :.

Xyr1_dom     648  ------DEFGDSPRAARGAHYECRGRSIFGYFLSLMTILGEIVDVHHAKSHPRFGVGFRS
Cep3p_dom    294  YRLCGCDYLQSGPRKPIALHTEVSSLLQHAAYLQDLPN----VDVYREEN---------S
Consensus    192  ------DELSPSSSPPPPPSEEPTSLSFFIALIRLARILGRILRLLYS---PRAS----S
                        * : ..   .     *  .   .   :         : :              *

Xyr1_dom     702  ARDWDEQVAEITRHLDMYEESLKRFVAKHLPLSSKDKEQHEMHDSGAVTDMQSPLSVRTN
Cep3p_dom    341  TEVLYWKIISLDRDLDQYLNKSSK-----PPLKTLDAIRRELDIFQYKVD-SLEEDFRSN
Consensus    239  PEDSLETILELDAELEEWRASL---------------------------------PPELRLD
                  ..     : .:  .*: :  .                                  ..* :

Xyr1_dom     762  ASSRMTESEIQASIVVAYSTHVMHVLH------ILLADKWDPINLLDDDDLWISSEGFVT
Cep3p_dom    395  NSRFQKFIALFQISTVSWKLFKMYLIYYDTADSLLKVIHYSKVIISLIVNNFHAKSEFFN
Consensus    268  SSSLESPSLLPQRLLL----HLLY--H------LLLILLHRPFLLRSLSSLSPSSSSAES
                   *   .   :     :    . ::  :      :*       . :    .   :..   .

Xyr1_dom     816  ATSHAVSAAEAISQILEF--DPGLEFMPFFYGVYLLQGSFLLLLIADKLQA------EAS
Cep3p_dom    455  RHPMVMQTITRVVSFISF------------YQIF-VESAAVKQLLVDLTELTANLPTIFG
Consensus    316  RRA-CLEAAREILELLRLLLSSSLLLLLWWFLLYYLFSAALVLLLALLLRPSSSELEEAR
                    .  :.:   : .:: :            : :: : .: :  *:.   .

Xyr1_dom     868  PSVIKACETIVRAHEACVVTLSTEYQRNFSKVMRSAL
Cep3p_dom    502  SKLDKLVYLTERLSKL---------------------
Consensus    375  SLLERALELLERLSERWP-RLARRALRLLERLLARAR
                  . : :      *  :
```
