# Supplementary material for: Mutation of the Xylanase regulator 1 causes a glucose blind hydrolase expressing phenotype in industrially used Trichoderma strains
Source: Biotechnol Biofuels. 2013 May 2;6:62. doi: 10.1186/1754-6834-6-62 (PMC3654998; doi:10.1186/1754-6834-6-62)
Supplement: Additional file 5 — Detailed protocol for cultivation in a bioreactor. [file 1754-6834-6-62-S5.pdf]

Additional file 5 - Detailed protocol for cultivation in a bioreactor

For cultivation in the bioreactor, spores from frozen (-80 oC) 15 %-glycerol stocks were inoculated onto standard 85 mm Petri plates containing potato dextrose agar (PDA). These plates were incubated at 28 °C for 3-5 days to achieve a confluent growth of fresh green spores. To prepare the inoculum for fermentation testing, spores from a single PDA plate were transferred to 2-L, baffled Erlenmeyer flasks containing 750 mL of liquid Berkley media (10.4 g/L (NH4)2SO4, 2.0 g/L KH2PO4, 0.31 g/L MgSO4·7H2O, 0.53 g/L CaCl2·2H2O, 1 mL/L of a trace elements solution containing 5 g/L FeSO4·7H2O; 1.6 g/L MnSO4·H2O; 1.4 g/L ZnSO4·7H2O,H, pH 5.5) supplemented with 5.1 g/L of corn steep liquor powder and 10 g/L glucose. Flasks were incubated at 28 °C for 3 days using an orbital agitator (Model G-52 New Brunswick Scientific Co.) running at 100 rpm. The contents of an inoculum flask were transferred to a 14 L pilot scale fermentation vessel (Model MF114 New Brunswick Scientific Co.) set up with 10 L of Initial Pilot Media (2.2 g/L (NH4)2SO4, 1.39 g/L KH2PO4, 0.7 g/L MgSO4·7H2O, 0.185 g/L CaCl2·2H2O, and 0.38 mL/L of a trace elements solution containing 5 g/L FeSO4·7H2O; 1.6 g/L MnSO4·H2O; 1.4 g/L ZnSO4·7H2O,H, pH 5.5) supplemented with 6 g/L of corn steep liquor powder and 13 g/L glucose. The vessel was run in batch mode until glucose in the media was depleted. At this point, a mixture of cellulase-inducing carbohydrates was added, from a stock that was 35.5% w/v of solids dissolved in water. Peristaltic pumps were used to deliver the carbon source at a feed at a rate of 0.4 g/L/h. Operational parameters during both the batch and fed-batch portions of the run were: mixing by impeller agitation at 500 rpm, air sparging at 8 standard L/min, and a temperature of 28 °C. Culture pH was maintained at 4.0 - 4.5 during batch growth and pH 5.0 during cellulase production using an automated controller connected to an online pH-probe and a pump enabling the addition of a 10 % ammonium hydroxide solution. Periodically, 100 mL samples of broth were drawn for biomass and protein analysis. The total fermentation time was 144 hours.
